# Management of immune checkpoint inhibitor‐related rheumatic adverse events

**DOI:** 10.1111/1759-7714.13249

**Published:** 2019-11-24

**Authors:** Jiaxin Zhou, Hanping Wang, Xiaoxiao Guo, Qian Wang, Lian Duan, Xiaoyan Si, Li Zhang, Xiaowei Liu, Yue Li, Wen Zhang, Li Zhang

**Affiliations:** ^1^ Department of Rheumatology Peking Union Medical College Hospital, Chinese Academy of Medical Sciences Beijing China; ^2^ Department of Respiratory and Critical Care Medicine Peking Union Medical College Hospital, Chinese Academy of Medical Sciences Beijing China; ^3^ Department of Cardiology Peking Union Medical College Hospital, Chinese Academy of Medical Sciences Beijing China; ^4^ Department of Endocrinology Peking Union Medical College Hospital, Peking Union Medical College and Chinese Academy of Medical Sciences Beijing China; ^5^ Department of Clinical Laboratory Peking Union Medical College Hospital, Peking Union Medical College and Chinese Academy of Medical Sciences Beijing China; ^6^ Department of Ophthalmology Peking Union Medical College Hospital, Peking Union Medical College and Chinese Academy of Medical Sciences Beijing China; ^7^ Department of Gastroenterology Peking Union Medical College Hospital, Peking Union Medical College and Chinese Academy of Medical Sciences Beijing China

**Keywords:** Immune checkpoint inhibitors, immune‐related adverse events, rheumatic disease, treatment

## Abstract

Immune checkpoint inhibitors (ICIs), which target the programmed cell death receptor‐1 and cytotoxic T lymphocyte‐associated antigen‐4 signaling pathways, represent remarkable breakthroughs in cancer treatment and have improved survival among patients with a variety of malignancies. However, the wide use of ICIs is associated with a spectrum of immune‐related adverse events (irAEs) that can affect any organ system, and may sometimes be life threatening. Rheumatic irAEs are not an infrequent type of irAE. In this systematic review, we consider the clinical characteristics of rheumatic irAEs, including patients with pre‐existing rheumatic diseases, and focus on the management of rheumatic irAEs.

## Introduction

Immune checkpoint inhibitors (ICIs) have recently been responsible for remarkable breakthroughs in cancer treatment. ICIs can induce T cell activation by blocking negative costimulation of T cells resulting in enhanced anti‐tumor effects, and can ultimately improve survival among patients with a variety of malignancies.^1^ Several ICIs targeting the programmed cell death receptor‐1 (PD‐1) and cytotoxic T lymphocyte‐associated antigen‐4 (CTLA‐4) pathways have been approved by the US Food and Drug Administration (FDA) and the European Medicines Agency.^2^, ^3^ However, ICIs can also affect the immune tolerance of human tissues, potentially leading to a new spectrum of adverse events, termed immune‐related adverse events (irAEs).^4^ irAEs can occur in almost every human organ system, and the clinical management of, and research into, irAEs thus involves oncologists and other medical specialists.

The major underlying mechanism of classical rheumatic autoimmune diseases involves abnormal activation of the immune system, leading to autoantibody formation or enhanced inflammatory responses. PD‐1 expression is increased in synovial tissues in patients with rheumatoid arthritis (RA), but the PD‐1 pathway is downregulated during RA disease progression, suggesting that this pathway might be involved in the development of RA.^5^ The CTLA‐4 pathway also plays an important role in the pathogenesis of RA, and abatacept, a fusion protein composed of CTLA‐4 and the Fc region of human immunoglobulin‐1, has already been approved by the FDA for the treatment of RA.^6^ Furthermore, the PD‐1 pathway has also been shown to be involved in preventing lupus‐like symptoms in mouse models.^7^

Rheumatic irAEs are not uncommon irAEs and can generally be classified into two subgroups: new‐onset musculoskeletal symptoms or connective tissue disease, and disease flares in patients with pre‐existing rheumatic conditions. In a large prospective French study of 524 patients who received ICIs,^8^ 35 patients (6.6%) developed rheumatic irAEs, including noninflammatory musculoskeletal symptoms, polymyalgia rheumatica (PMR), and RA. The median period between ICI exposure and the occurrence of rheumatic irAEs was 70 days. Arthralgia and myalgia were the most common symptoms of rheumatic irAEs. However, these symptoms may be overlooked in research and clinical practice if their symptoms are mild, and it is therefore necessary to remind clinicians about the possibility and importance of rheumatic irAEs.

## Polymyalgia rheumatica (PMR)/giant cell arteritis (GCA)

Polymyalgia rheumatica (PMR) is an inflammatory disease commonly seen in individuals older than 50 years. It is characterized by shoulder and/or pelvic girdle muscle myalgia and stiffness, with increased acute phase reactants and negative rheumatoid factors or anti‐citrullinated protein antibody (ACPA). This disorder always responds well to low‐dose glucocorticoids. Giant cell arthritis (GCA) is a type of systemic vasculitis that is relatively rare in Chinese patients, but which has a very close relationship with PMR. GCA is characterized by large‐vessel involvement with inflammation of the arterial wall, and involvement of the internal elastic lamina and multinucleated giant cell infiltration.^9^

The median period from ICI exposure to PMR occurrence varies from 10 days to one year, with similar clinical and radiological manifestations compared with classical PMR. However, patients with ICI‐induced PMR do not always have increased acute phase reactants and may not respond well to low‐dose glucocorticoids.^10^ A few patients may develop GCA after ICI treatment, with clinical symptoms including headache, temporal artery tenderness, jaw claudication, and vision loss, and pathological manifestations similar to classical GCA.^11^, ^12^

## Inflammatory arthritis

ICI‐induced inflammatory arthritis is one of the most common rheumatic irAEs and has been previously reported in several studies. The median period from ICI treatment to inflammatory arthritis onset ranges from two months to two years. Moreover, ICI‐related inflammatory arthritis varies in severity from mild disease, which responds well to nonsteroidal anti‐inflammatory drugs (NSAIDs) or low‐dose glucocorticoids, to severe cases that may require tumor necrosis factor (TNF) inhibitors or interleukin‐6 (IL‐6) receptor antibodies to ameliorate the symptoms.^13^, ^14^, ^15^ ICI‐related inflammatory arthritis can be further classified into two subgroups. The first type of IA is similar to RA, involving the small joints (proximal interphalangeal joints, metacarpophalangeal joints, and wrists) and bone erosions. However unlike RA, these patients do not show a female predominance and are usually seronegative for rheumatoid factor and ACPA, with earlier bone erosions; however, a few patients with ACPA‐positive RA after ICI treatment were also found to be seropositive for ACPA before treatment with ICIs. The second group of patients had clinical manifestations similar to spondyloarthritis, including inflammatory back pain, enthesitis, dactylitis, and, oligoarthritis with predominantly large‐joint involvement. A small subset of patients may manifest as reactive arthritis or psoriatic arthritis. However, HLA‐B27 positivity was rarely seen in the second group of patients.^10^, ^16^

## Inflammatory myopathy

ICI‐induced inflammatory myopathy is relatively less common than inflammatory arthritis and always occurs in the first two months after ICI treatment, with sudden onset. Inflammatory myopathy is characterized by myalgia, muscle weakness, and elevated serum creatinine kinase (CK) levels. Severe cases may include rhabdomyolysis, respiratory muscle weakness, or myocardial involvement, and the condition can sometimes be life‐threatening. Muscle biopsies show focal necrotizing myopathy with extensive macrophage and T lymphocyte infiltration. Perifascicular atrophy of muscle fibers, which is a classic pathological finding of dermatomyositis, has not been found in ICI‐induced inflammatory myopathy. ICI‐related inflammatory myopathy also rarely presents with a classical dermatomyositis‐related rash, and patients are seronegative for myositis‐specific antibodies and may also have involvement of the axial part of the facial muscle.^17^, ^18^, ^19^, ^20^, ^21^, ^22^ Concomitant inflammatory fasciitis or myasthenia gravis can also occasionally occur in patients with ICI‐induced inflammatory myopathy.^23^, ^24^ In our clinic, we have experienced several patients with markedly elevated serum CK levels but no obvious clinical symptoms such as myalgia or muscle weakness. This highlights the need to routinely monitor serum CK levels in patients taking ICIs, even if they have no complaints of myalgia or muscle weakness.

## Other connective tissue diseases

New‐onset connective tissue disease after ICI treatment is rare. A study at Johns Hopkins Hospital of patients with solid tumors who received ipilimumab and/or nivolumab treatment from 2012 to 2016 included four patients who developed sicca syndrome with relatively abrupt onset of severe dry mouth symptoms, one patient with bilateral parotid gland enlargement, and two with other irAEs. None of these patients had anti‐Ro/SSA antibody.^14^ In the report by Ghosn *et al*., a patient with melanoma treated with pembrolizumab developed typical Sjögren syndrome with subacute ataxic sensory neuropathy with only a partial response to glucocorticoids, but demonstrated remarkable improvement after treatment with intravenous immunoglobulins and rituximab.^25^

Barbosa *et al*. reported on two patients with melanoma and newly developed systemic sclerosis after pembrolizumab treatment.^26^ There have also been occasional reports of GCA, single‐organ vasculitis, and granulomatosis with polyangiitis.^27^, ^28^, ^29^

## Patients with pre‐existing rheumatic diseases

There is limited information about flares of pre‐existing rheumatic diseases in patients treated with ICIs because such patients have tended to be excluded from clinical trials. However, we managed a patient with gastric cancer who was also diagnosed with connective tissue disease associated interstitial lung disease. They received three cycles of nivulomab therapy and had a high fever and rapid progression of interstitial lung disease, which failed to respond to medium‐dose glucocorticoids but was well‐controlled by high‐dose glucocorticoids and tacrolimus. A retrospective study conducted at Massachusetts General Hospital reported six patients with rheumatic disease flares after ICI treatment,^15^ including one case of RA, two of PMR, two of RA with PMR, and one of psoriatic arthritis. The median time from ICI exposure to rheumatic disease flare was 4.6 weeks, and one severe case was finally controlled using tocilizumab. Mitchell *et al* 20 reported an Australian study including 12 patients with underlying rheumatic diseases, of whom 10 experienced disease flares after ICI therapy, including four cases of inflammatory arthritis and six of PMR. However, patients with rheumatic diseases can also develop new‐onset irAEs after ICI treatment. Among 700 patients treated with ICIs and reviewed in a study by the Mayo Clinic,^30^ 16 had a past history of rheumatic diseases. Six patients with rheumatic diseases developed irAEs after ICI treatment, but only one patient with GCA had a disease flare, while the other five patients all had newly occurring irAEs, such as colitis and hypophysitis. It is therefore important to be aware of the possibility of disease flares after ICI treatment in patients with pre‐existing rheumatic diseases, and for clinicians to make a differential diagnosis between rheumatic disease flares and new‐onset irAEs.

## Management of ICI‐induced rheumatic irAEs

It is important for rheumatologists to collaborate with oncologists to manage patients with rheumatic irAEs, including the decision on whether or not to continue ICI treatment. Considering that patients with rheumatic irAEs tended to have better cancer‐treatment outcomes,^8^ ICIs should generally be continued if they show positive results. However, ICIs should be temporally or even permanently stopped in the event of severe irAEs.^10^ In addition, more large prospective studies are needed to determine if glucocorticoids or immunosuppressants affect the anti‐tumor effects of ICIs.

NSAIDs are the first line of therapy in patients with inflammatory arthritis with mild symptoms, and low‐dose glucocorticoids (prednisone 10–20 mg/day or equivalent dose) can be considered, while NSAIDs are less effective. For patients with moderately severe inflammatory arthritis, prednisone >20 mg/day or equivalent dose plus disease‐modifying anti‐rheumatic drug (DMARDs) therapy is recommended, while the dose of glucocorticoids can be further increased in cases of severe inflammatory arthritis, and biological agents such as TNF inhibitors can also be used in glucocorticoid‐dependent patients.^31^, ^32^ The use of TNF inhibitors is generally contraindicated in patients with malignancies; however, considering the risks and urgency of tumor patients complicated with irAEs, TNF inhibitors are still an option for managing severe inflammatory arthritis. Perez‐Ruiz *et al*. recently reported that the prophylactic use of TNF inhibitors concomitant with combined anti‐CTLA‐4 and anti‐PD‐1 therapy in mouse models ameliorated irAEs and also improved anti‐tumor efficacy.^33^ It is therefore important to understand the role of TNF inhibitors in tumor immunotherapy. Moreover, one report described a patient with severe arthritis after ICI treatment that was controlled by IL‐6 receptor antibody (tocilizumab) treatment.^34^ However, the effectiveness of other biological agents such as IL‐17 and IL‐12/23 antagonists are still under investigation.

ICI treatment can be continued in patients with mild myalgia, while serum CK levels should be monitored closely and NSAIDs can be used to control the myalgia. Serum CK levels usually decrease gradually after cessation of immunotherapy in patients with mild to moderate elevation of serum CK levels, but without myalgia or muscle weakness. However, in patients with moderate to severe myositis, electromyograms, muscle magnetic resonance imaging, myositis‐specific autoantibody testing, and muscle biopsy should be performed to differentiate between irAE and classical myositis. Glucocorticoids are the first‐line therapy for ICI‐induced myositis, with an initial dose of 0.5–1.0 mg/kg/day prednisone according to the severity of muscle involvement. Intravenous methylprednisolone pulse therapy should be considered and possibly combined with intravenous immunoglobulins in patients with myocardial involvement or life‐threatening conditions, and DAMRDs should be used in glucocorticoid‐dependent patients.^18^, ^19^, ^22^, ^32^ Salem *et al*. recently reported a case in which the CTLA‐4 agonist abatacept was successfully used to treat severe and glucocorticoid‐refractory ICI‐induced myocarditis, offering a new approach for the treatment of patients with severe ICI‐induced myocarditis.^35^ However, further studies are needed to evaluate the risks and benefits of abatacept more thoroughly in these patients.

ICI should be stopped in patients with pre‐existing rheumatic diseases who experience flare‐ups after ICI treatment. Subsequent treatment should then follow the management recommendations for classical rheumatic diseases which usually base the glucocorticoid and immunosuppressant doses on the type and severity of organ involvement.

## Conclusions

ICIs represent a new era in the treatment of malignancies, although the concomitant irAEs, including rheumatic irAEs, also bring new challenges and research areas for clinicians. Rheumatologists must engage more in the management and basic research of irAEs.

## Disclosure

The authors have no potential conflicts of interest to disclose.

## References

[1] Cappelli LC , Gutierrez AK , Bingham CO 3rd *et al* Rheumatic and musculoskeletal immune‐related adverse events due to immune checkpoint inhibitors: A systematic review of the literature. Arthritis Care Res (Hoboken) 2017; 69: 1751–63. 10.1002/acr.23177. 27998041 PMC5478477

[2] Hoos A . Development of immuno‐oncology drugs ‐ from CTLA4 to PD1 to the next generations. Nat Rev Drug Discov 2016; 15: 235–47. 10.1038/nrd.2015.35. 26965203

[3] Postow MA , Callahan MK , Wolchok JD . Immune checkpoint blockade in cancer therapy. J Clin Oncol 2015; 33: 1974–82. 10.1200/JCO.2014.59.4358. 25605845 PMC4980573

[4] Postow MA , Sidlow R , Hellmann MD . Immune‐related adverse events associated with immune checkpoint blockade. N Engl J Med 2018; 378: 158–68. 10.1056/NEJMra1703481. 29320654

[5] Guo Y , Walsh AM , Canavan M *et al* Immune checkpoint inhibitor PD‐1 pathway is down‐regulated in synovium at various stages of rheumatoid arthritis disease progression. PLOS One 2018; 13: e0192704 10.1371/journal.pone.0192704. 29489833 PMC5831027

[6] Blair HA , Deeks ED . Abatacept: A review in rheumatoid arthritis. Drugs 2017; 77: 1221–33. 10.1007/s40265-017-0775-4. 28608166

[7] Nishimura H , Nose M , Hiai H , Minato N , Honjo T . Development of lupus‐like autoimmune diseases by disruption of the PD‐1 gene encoding an ITIM motif‐carrying immunoreceptor. Immunity 1999; 11: 141–51. 10.1016/S1074-7613(00)80089-8. 10485649

[8] Kostine M , Rouxel L , Barnetche T *et al* Rheumatic disorders associated with immune checkpoint inhibitors in patients with cancer‐clinical aspects and relationship with tumour response: A single‐Centre prospective cohort study. Ann Rheum Dis 2018; 77: 393–8. 10.1136/annrheumdis-2017-212257. 29146737

[9] Chinese Rheumatology Association . The guideline for the diagnosis and treatment of polymyalgia rheumatica and giant cell arteritis. Chin J Rheumatol 2011; 15: 348–50. 10.3760/cma.j.issn.1007-7480.2011.05.016.

[10] Calabrese LH , Calabrese C , Cappelli LC . Rheumatic immune‐related adverse events from cancer immunotherapy. Nat Rev Rheumatol 2018; 14: 569–79. 10.1038/s41584-018-0074-9. 30171203

[11] Goldstein BL , Gedmintas L , Todd DJ . Drug‐associated polymyalgia rheumatica/giant cell arteritis occurring in two patients after treatment with ipilimumab, an antagonist of ctla‐4. Arthritis Rheumatol 2014; 66: 768–9. 10.1002/art.38282. 24574239

[12] Micaily I , Chernoff M . An unknown reaction to pembrolizumab: Giant cell arteritis. Ann Oncol 2017; 28: 2621–2. 10.1093/annonc/mdx306. 28633343

[13] Lidar M , Giat E , Garelick D *et al* Rheumatic manifestations among cancer patients treated with immune checkpoint inhibitors. Autoimmun Rev 2018; 17: 284–9. 10.1016/j.autrev.2018.01.003. 29341936

[14] Cappelli LC , Gutierrez AK , Baer AN *et al* Inflammatory arthritis and sicca syndrome induced by nivolumab and ipilimumab. Ann Rheum Dis 2017; 76: 43–50. 10.1136/annrheumdis-2016-209595. 27307501 PMC5333990

[15] Mooradian MJ , Nasrallah M , Gainor JF *et al* Musculoskeletal rheumatic complications of immune checkpoint inhibitor therapy: A single center experience. Semin Arthritis Rheum 2019; 48: 1127–32. 10.1016/j.semarthrit.2018.10.012. 30409415

[16] Lee KA , Kim HR , Yoon SY . Rheumatic complications in cancer patients treated with immune checkpoint inhibitors. Korean J Intern Med 2019; 34: 1197–1209. 10.3904/kjim.2019.060 [Epub ahead of print]. 31014065 PMC6823575

[17] Liewluck T , Kao JC , Mauermann ML . PD‐1 inhibitor‐associated myopathies: Emerging immune‐mediated myopathies. J Immunother 2018; 41: 208–11. 10.1097/CJI.0000000000000196. 29200081

[18] Shah M , Tayar JH , Abdel‐Wahab N , Suarez‐Almazor ME . Myositis as an adverse event of immune checkpoint blockade for cancer therapy. Semin Arthritis Rheum 2019; 48: 736–40. 10.1016/j.semarthrit.2018.05.006. 29909921

[19] Kadota H , Gono T , Shirai Y , Okazaki Y , Takeno M , Kuwana M . Immune checkpoint inhibitor‐induced myositis: A case report and literature review. Curr Rheumatol Rep 2019; 21: 10 10.1007/s11926-019-0811-3. 30790071

[20] Mitchell EL , Lau PKH , Khoo C *et al* Rheumatic immune‐related adverse events secondary to anti‐programmed death‐1 antibodies and preliminary analysis on the impact of corticosteroids on anti‐tumour response: A case series. Eur J Cancer 2018; 105: 88–102. 10.1016/j.ejca.2018.09.027. 30439628

[21] Tauber M , Cohen R , Laly P , Josselin L , André T , Mekinian A . Severe necrotizing myositis associated with long term anti‐neoplastic efficacy following nivolumab plus ipilimumab combination therapy. Clin Rheumatol 2019; 38: 601–2. 10.1007/s10067-018-4373-y. 30456528

[22] Ganatra S , Neilan TG . Immune checkpoint inhibitor‐associated myocarditis. Oncologist 2018; 23: 879–86. 10.1634/theoncologist.2018-0130. 29802219 PMC6156176

[23] Narvaez J , Juarez‐Lopez P , Lluch J *et al* Rheumatic immune‐related adverse events in patients on anti‐PD‐1 inhibitors: Fasciitis with myositis syndrome as a new complication of immunotherapy. Autoimmun Rev 2018; 17: 1040–5. 10.1016/j.autrev.2018.05.002. 30103042

[24] Makarious D , Horwood K , Coward JIG . Myasthenia gravis: An emerging toxicity of immune checkpoint inhibitors. Eur J Cancer 2017; 82: 128–36. 10.1016/j.ejca.2017.05.041. 28666240

[25] Ghosn J , Vicino A , Michielin O , Coukos G , Kuntzer T , Obeid M . A severe case of neuro‐Sjögren's syndrome induced by pembrolizumab. J Immunother Cancer 2018; 6: 110 10.1186/s40425-018-0429-4. 30348223 PMC6196470

[26] Barbosa NS , Wetter DA , Wieland CN , Shenoy NK , Markovic SN , Thanarajasingam U . Scleroderma induced by pembrolizumab: A case series. Mayo Clin Proc 2017; 92: 1158–63. 10.1016/j.mayocp.2017.03.016. 28599746

[27] Läubli H , Hench J , Stanczak M *et al* Cerebral vasculitis mimicking intracranial metastatic progression of lung cancer during PD‐1 blockade. J Immunother Cancer 2017; 5: 46 10.1186/s40425-017-0249-y. 28642817 PMC5477093

[28] Manusow JS , Khoja L , Pesin N , Joshua AM , Mandelcorn ED . Retinal vasculitis and ocular vitreous metastasis following complete response to PD‐1 inhibition in a patient with metastatic cutaneous melanoma. J Immunother Cancer 2014; 2: 41 10.1186/s40425-014-0041-1. 25516805 PMC4266968

[29] Rutgers A , van den Brom RRH , Hospers GAP , Heeringa P , Brouwer E . Systemic vasculitis developed after immune checkpoint inhibition. Arthritis Care Res (Hoboken) 2018; 70: 1275 10.1002/acr.23481. 29195007

[30] Richter MD , Pinkston O , Kottschade LA , Finnes HD , Markovic SN , Thanarajasingam U . Brief report: Cancer immunotherapy in patients with preexisting rheumatic disease: The Mayo Clinic experience. Arthritis Rheumatol 2018; 70: 356–60. 10.1002/art.40397. 29363290

[31] Trinh S , Le A , Gowani S *et al* Management of immune‐related adverse events associated with immune checkpoint inhibitor therapy: A mini review of current clinical guidelines. Asia Pac J Oncol Nurs 2019; 6: 154–60. 10.4103/apjon.apjon_3_19. 30931360 PMC6371672

[32] Thompson JA , Schneider BJ , Brahmer J *et al* Management of immunotherapy‐related toxicities, version 1.2019. J Natl Compr Canc Netw 2019; 17: 255–89. 10.6004/jnccn.2019.0013. 30865922

[33] Perez‐Ruiz E , Minute L , Otano I *et al* Prophylactic TNF blockade uncouples efficacy and toxicity in dual CTLA‐4 and PD‐1 immunotherapy. Nature 2019; 569: 428–32. 10.1038/s41586-019-1162-y. 31043740

[34] Kim ST , Tayar J , Trinh VA *et al* Successful treatment of arthritis induced by checkpoint inhibitors with tocilizumab: A case series. Ann Rheum Dis 2017; 76: 2061–4. 10.1136/annrheumdis-2017-211560. 28830882

[35] Salem JE , Allenbach Y , Vozy A *et al* Abatacept for severe immune checkpoint inhibitor‐associated myocarditis. N Engl J Med 2019; 380: 2377–9. 10.1056/NEJMc1901677. 31189043

